# Atopobiosis and Dysbiosis in Ocular Diseases: Is Fecal Microbiota Transplant and Probiotics a Promising Solution?

**DOI:** 10.18502/jovr.v16i4.9754

**Published:** 2021-10-25

**Authors:** Triana Hardianti Gunardi, Diannisa Paramita Susantono, Andi Arus Victor, Ratna Sitompul

**Affiliations:** ^1^Faculty of Medicine, Universitas Indonesia, Jakarta, Indonesia; ^2^Department of Ophthalmology, Dr. Cipto Mangunkusumo National General Hospital – Faculty of Medicine, Universitas Indonesia, Jakarta, Indonesia

**Keywords:** Atopobiosis, Autoimmune, Dysbiosis, Gut–Eye Axis, Uveitis

## Abstract

**Purpose:**

To highlight the role of atopobiosis and dysbiosis in the pathomechanism of autoimmune uveitis, therefore supporting fecal microbiota transplant (FMT) and probiotics as potential targeted-treatment for uveitis.

**Methods:**

This review synthesized literatures upon the relation between gut microbiota, autoimmune uveitis, FMT, and probiotics, published from January 2001 to March 2021 and indexed in PubMed, Google Scholar, CrossRef.

**Results:**

The basis of the gut–eye axis revolves around occurrences of molecular mimicry, increase in pro-inflammatory cytokines, gut epithelial barrier disruption, and translocation of microbes to distant sites. In patients with autoimmune uveitis, an increase of gut *Fusobacterium* and *Enterobacterium* were found. With current knowledge of aforementioned mechanisms, studies modifying the gut microbiome and restoring the physiologic gut barrier has been the main focus for pathomechanism-based therapy. In mice models, FMT and probiotics targeting repopulation of gut microbiota has shown significant improvement in clinical manifestations of uveitis. Consequently, a better understanding in the homeostasis of gut microbiome along with their role in the gut–eye axis is needed to develop practical targeted treatment.

**Conclusion:**

Current preliminary studies are promising in establishing a causative gut–eye axis relationship and the possibility of conducting FMT and probiotics as targeted treatment to mitigate autoimmune uveitis, to shorten disease duration, and to prevent further complications.

##  INTRODUCTION

Humans require commensal microorganisms to carry out vital bodily functions.^[1]^ These microorganisms (in groups: *microbiome*) live most abundantly within the human gut; and are heavily influenced by internal (age, race, ethnicity, gender, genetics) as well as external factors (diet, consumption of antibiotics, sanitation, geographic domicile).^[1,2]^ The perspective of viewing the human body as a vast interchangeable ecosystem, alters how medicine works in practice.^[1]^ Instead of treating microbiome as a harmful target, we recognize the efficacy of nurturing and repopulating it to its homeostatic state within human body.^[2]^ Many studies have reported on how its altered composition may induce systemic immune response, hematogenic spread, and even translocation of microbes to distant sites.^[1]^ Focusing on preventing these mechanisms, is expected to provide an alternative solution to autoimmune diseases.

Among the autoimmune diseases with ocular involvement, uveitis is one of the most complex and is accountable for 25% blindness in the world.^[3]^ It may manifest as a localized or part of a systemic disease. Autoimmune diseases in relation to the eyes can be divided into: (a) systemic diseases with ocular manifestations, such as Sarcoidosis, Behcet's disease, multiple sclerosis, systemic lupus erythematosus (SLE), rheumatoid arthritis, ocular cicatrical pemphigoid, Sjögren syndrome; (b) localized autoimmune ocular diseases, such as sympathetic ophthalmia, birdshot retinochoroidopathy, and Mooren's ulcerative keratitis.^[4]^ Current treatments for uveitis include topical and systemic anti-inflammatory drugs (nonsteroidal anti-inflammatory drugs and corticosteroids), and immunomodulation-based therapies in severe cases.^[5]^ Effective targeted therapies are yet to be discovered. Available data on gut microbiota in association with uveitis or ocular diseases in humans are still very limited. This review summarized the current knowledge on the role of gut microbiota in uveitis through atopobiosis and dysbiosis mechanisms, followed by a proposed alternative concept for potential uveitis-targeted treatment.

##  METHODS

### Literature Search Strategy

We performed a systematic literature search using electronic database: PubMed, Google Scholar, CrossRef; using the following keywords, “atopobiosis” or “dysbiosis” or “gut microbiota” or “gut microbiome” and “ocular diseases” or “autoimmune diseases” or “immune-related diseases” or “uveitis” or “autoimmune uveitis” and “fecal microbiota transplant” or “probiotics” or “treatment” or “therapy”.

We focused on two main purposes: (1) summarizing the current knowledge on the role of gut microbiota in uveitis through atopobiosis and dysbiosis mechanism; (2) proposing an alternative concept for potential uveitis-targeted treatment. Articles discussing dysbiosis and/or atopobiosis in other ocular diseases were included as supporting evidence of the gut–eye axis.

### Eligibility Criteria

All accessible full articles, published from January 2001 to August 2020 were included. Duplicates were omitted. We retrieved 115 articles from selected database, followed by exclusion of doubles and non-accessible full papers, yielding 56 articles which have undergone thorough review to be summarized.

##  RESULTS 

### The Gut Microbiome, Atopobiosis, and Dysbiosis

The microbiome is spread throughout the human body. They work in a bidirectional relationship with the host's immune system, creating balance between pro-inflammatory (e.g., Th1, Th17) and anti-inflammatory (e.g., Treg) mechanisms. As demonstrated in mice models, segmented filamentous bacteria promote pro-inflammatory Th1 and Th17 cells in the lamina propria, while Treg cells facilitate anti-inflammatory response through short-chain-fatty-acid production.^[6,7]^ *Bacteroides fragilis* and *Faecalibacterium prausnitzii* promote the accumulation of Treg cells.^[8,9]^ In human adults, *Bacteroides spp.* (gram-negative bacteria) and *Firmicutes* (gram-positive bacteria) usually predominate > 93% of the gut microbial population. The rest is occupied by minor constituents.^[10,11]^ Both major and minor constituents exert significant impact on the microbiome homeostasis. Table 1 illustrates the taxonomic gut microbiota.^[10]^

The 16S rRNA assays and shotgun metagenomics have been utilized in characterizing gut microbiome composition, up to genus and species, respectively, and also its alteration by controlled interventions.^[12]^ These assays allow identification of microbes that cannot be cultured, due to exiguous numbers or peculiar conditions.^[13,14,15]^ For example, there is an increase of sulphate-reducing bacteria has been found in the feces of ankylosing spondylitis patients compared to healthy controls of identical age and gender. Meanwhile, there is an increase of *Bacterodales*, decrease of *Firmicutes*/*Bacteroidetes* ratio, and a reduction of *Lachnospiraceae* and *Ruminococcaceae* within the fecal microbiome profile of SLE patients compared to the healthy control group.^[16,17]^ Although no standard or “normal” microbiome ratio has ever been established, evidence suggests shifts in the microbiome has a significant impact on host immune system.

Dysbiosis is the shifting of microbiome composition into a pathogenic state, while atopobiosis is the translocation of microbes to places other than their normal location.^[18]^ The two conditions have been recognized in the pathogenesis of multiple immune-mediated inflammatory diseases, such as rheumatoid arthritis, inflammatory bowel disease, and multiple sclerosis.^[13]^ Gut dysbiosis is particularly established to play a role in altering Treg–Th17 balance by causing a Th17 expansion, thus signaling the release of pro-inflammatory cytokines (IL-6, IL-17, IL-21, IL-23, IFN- γ ) in the gut lamina propria.^[14]^ This pro-inflammatory state suppresses tight junction proteins (occludin and claudin), causing increased permeability of the lamina propria allowing antigen exposure from the gut microbiome. These contents from the gut microbiome may be presented as antigens to the Antigen Presenting Cell (APC)s; or in the case of atopobiosis, the microbes may translocate to other sites.^[14,18]^ Whichever pathway they were taken into, the process continues as cytokines and other inflammatory-mediators activate T-cells, B-cells, and dendritic-cells from the gut, which then travel through the lymphatic drainage to the mesenteric lymph nodes. Antigen presentation and cell differentiation proceeds, leading to the production of activated B-cells, Th17-cells, and plasma cells. Within this cascade, gut microbes are suspected to play role as mimicry antigens leading to the stimulation of autoreactive T-cells and B-cells.^[6,14]^ In many autoimmune diseases, presence of these autoreactive immune cells and/or translocation of the pathogen to target organs leads to inflammatory reaction, thus it has become the basis of multiple gut-*organ* axis hypotheses (e.g., gut–brain axis, gut–joint axis).^[18]^ Autoimmune uveitis is hypothesized to be an inflammatory reaction following the aforementioned cascade.^[13][14,18][19][20]^

**Figure 1:**
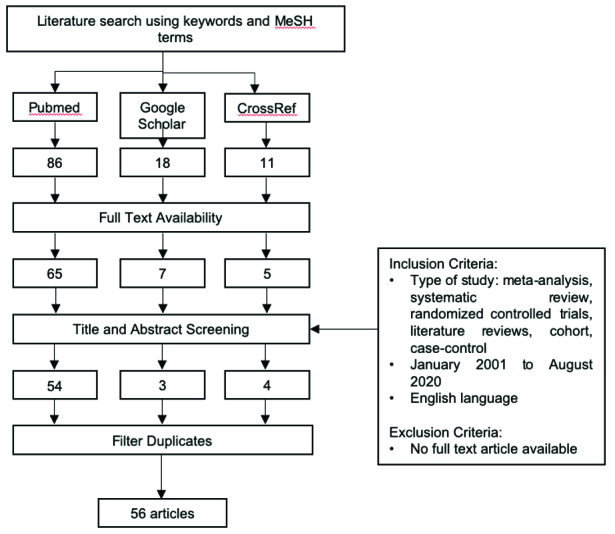
Flowchart for study selection process.

**Figure 2:**
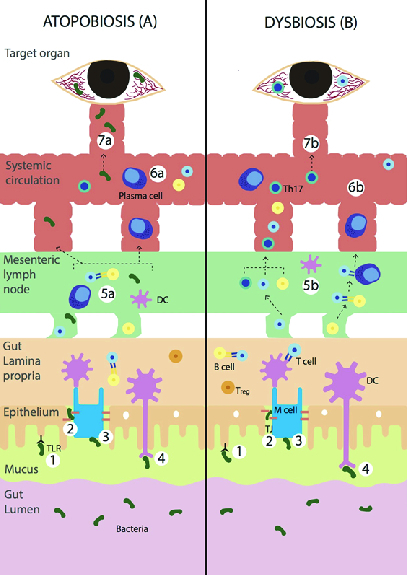
Illustrated pathogenesis of atopobiosis and dysbiosis in relation to uveitis. (1) Microbe recognition. (2) Bypass via tight junction. (3) Transcytosis via M-cells. (4) Phagocytosis by DCs and APCs. (5a) Microbes or bacteria enter the mesenteric lymph node. (6a) Bacteria enter the systemic circulation. (7a) Translocation of bacteria to ocular endothelial site. (5b) DCs, T cells, B cells enter the mesenteric lymph nodes. (6b) These cells may undergo cascades and enter the systemic circulation. (7b) Activated retina specific T-cells surpass the blood–retinal barrier causing inflammation. TLR, toll-like receptor; DC, dendritic cell; APC, antigen presenting cell; Th17, T helper

**Table 1:** Taxonomic terminology of the human gut microbiota


**Phylum**	**Class**	**Genus**	**Species**
*Actinobacteria*	Actinomycetales	*Corynebacterium*	
	Bifidobacteriales	*Bifidobacterium*	*Bifidobacterium longum*
		*Bifidobacterium bifidum*
	*Coriobacteriia*	*Atopobium*	
*Firmicutes*	*Clostridia*	*Faecalibacterium*	*Faecalibacterium prausnitzii*
	*Clostridium*	*Clostridium* *spp.*
	*Roseburia*	*Roseburia intestinalis*
	*Ruminococcus*	*Ruminococcus faecis*
	Negativicutes	*Dialister*	*Dialister invisus*
	Bacilli	*Lactobacillus*	*Lactobacillus reuteri*
	*Enterococcus*	*Enterococcus faecium*
	*Staphylococcus*	*Staphylococcus leei*
*Bacteroidetes*	*Sphingobacteriia*	*Sphingobacterium*	
	*Bacteroidia*	*Bacteroides*	*Bacteroides fragilis*
		*Bacteroides vulgatus*
		*Bacteroides uniformis*
	*Tannerella*	
	*Parabacteroides*	*Parabacteroides distasonis*
	*Alistipes*	*Alistipes finegoldii*
	*Prevotella*	*Prevotella spp.*
Verrucomicrobia	Verrucomicrobia	*Akkermansia*	*Akkermansia muciniphila*
Fusobacteria	Fusobacteriia	*Fusobacterium*	*Fusobacterium nucleatum*
Proteobacteria	Gamma proteobacteria	*Escherichia*	*Eschericia coli*
	*Shigella*	*Shieflla flexneri*
	Delta proteobacteria	*Desulfovibrio*	*Desulfovibrio intestinales*
	*Bilophila*	*Bilophila wadsworthia*
	Epsilon proteobacteria	*Helicobacter*	*Helicobacter pylori*
The *Firmicutes* and *Bacteroidetes* predominant the gut microbiome in human^[11]^			

**Table 2:** Gut microbiota proportions in ocular diseases


**Disease**	**Findings**
Dry eye in Sjögren's	Mice: increased numbers of *Enterobacter, Escherichia/Shigella, Pseudomonas, *and decreased numbers of *Clostridium.* ^[52]^ Human: increased numbers of Bacteroides*, Parabacteroides*, Actinobacteria, Pseudobutyrivibrio, Escherichia/Shigella, Blautia, Streptococcus, anddecreased numbers of* Firmicutes, Faecalibacterium, Prevotella, Viellonella.* ^[52,53]^
Uveitis	Mice: decreased numbers of *Rikenellaceae* and increased numbers of *Paraprevotella*.^[25]^ Human: increased numbers of *Fusobacterium* and *Enterobacteriaceace*.^[51]^
Diabetic retinopathy	Mice: increased numbers of *Firmicutes* and decreased *Bacteroidetes*.^[54]^ Human: decreased numbers of *Bacteroides* and *Lactobacillus.* ^[55,56]^
Age-related macular degeneration	Mice: increased numbers of *Firmicutes* and *Clostridia*, and decreased numbers of *Bacteroidetes* and *Erysipelotrichi*.^[30]^ Human: increased numbers of *Ruminococcaceae,* *Prevotella, Anaerotruncus, Oscillibacter, Ruminiococcus torques*, and *Eubacterium ventriosum*.^[51,57]^
Bacterial keratitis	Human: increased numbers of *Proteobacteria* and *Firmicutes*.^[58]^
*There were discrepancies between studies	

The relationship between autoimmune uveitis with gut dysbiosis has been demonstrated in multiple studies, whereas its association with atopobiosis is less established. A recent study by Deng et al has revealed that the widely accepted to be sterile - aqueous humor has shown microbial presence. These findings were found in patients with AMD and glaucoma, in which disease-specific microbial signatures were found.^[21]^ Gómez et al also reported microbiome translocation from periodontal infection to placenta. *Porphyromonas gingivalis *from pregnant mothers could translocate to the placenta, thus activating inflammatory response of the decidual tissue. It created a switch of the Th-1 profile balance toward an inflammatory state, mediated by monocyte chemoattractant protein-1 (MCP-1) and macrophages.^[22]^ These mechanisms manifested clinically as adverse pregnancy outcomes (APOs). The APOs observed included low birth weight, preterm premature rupture of membranes, preterm birth, and other clinical signs related to chorioamnionitis.^[22]^ These findings suggest microbiome translocation and possibly a hematogenic spread via the blood brain barrier and/or placental circulation. Further studies are imperative to investigate the intraocular microbiome profile in uveitis and the corresponding gut microbiome.

### Early Evidence of the Gut–Eye Axis: A Look into Microbiome Shift in Autoimmune Uveitis

Microbiome alterations have been found in ocular diseases including dry eyes, uveitis, diabetic retinopathy, age-related macular degeneration (AMD), etc. [Table 2].^[1,2][19][23]^ Among the evidence supporting the gut–eye axis, its correlation with autoimmune uveitis is one of the most heavily studied. Uveitis is a complex inflammation of the eye, a manifestation of > 30 different etiologies, including infectious and autoimmune origins.^[24]^

Characterization of the gut microbiota in patients with uveitis has been done in both animal models and humans. Lin et al studied transgenic mice carrying the HLA-B27 gene, a major risk factor for acute anterior uveitis. They found increased numbers of *Paraprevotella* and *Bacteroides vulgatus, *and decrease of *Rikenellaceae *in HLA-B27 mice compared to wild-type mice.^[25]^ Characterization of gut microbiota in humans with Behçet's disease revealed an altered gut microbiota composition with reduced butylate production and increased fecal secretory IgA.^[26,27]^ Ye et al revealed an increased level of *Bilophila spp., Parabacteroides spp., Paraprevotella spp., *and decreased level of* Clostridium spp., Methanocelleus spp., Methanomethylophilus spp. *in the gut of Behçet's disease patients. Kalyana Chakravarthy et al studied the gut composition of patients with *Vogt-Koyanagi-Harada* and idiopathic uveitis. They found decreased level of *Faecalibacterium, Bacteroides, Lachnospira, Ruminococcus, *and enrichment of *Prevotella* and *Streptococcus*. Meanwhile the gut fungal microbiome profile showed increased numbers of pathogenic fungi including *Malassezia restricta, Candida albicans, Candida glabrata, Aspergillus gracilis,* compared to healthy controls.^[28]^ There were no significant differences were seen in the microbiome profile of autoimmune uveitis compared to idiopathic uveitis, suggesting both were influenced by dysbiosis.^[3]^

Further association of the gut microbiota's role in manifestation of uveitis was demonstrated using the B10.RIII mice model which develop uveitis when injected with interphotoreceptor retinoid binding protein (IRBP) antigens.^[29]^ Nakamura et al intervened with the gut composition of this mice model using oral broad-spectrum antibiotics (ampicillin, metronidazole, neomycin, vancomycin) one week before inducing uveitis. They compared them to B10.RIII mice that had not received antibiotics. The antibiotic-treated mice showed reduced severity of uveitis.^[29]^ Singular antibiotic administration in this model revealed significant signs of uveitis only when given metronidazole or vancomycin, meanwhile administration of ampicillin or neomycin did not.^[29]^ These conditions provide a clue on the particular groups of microbes playing a role in this pathway. The mouse model when treated with oral broad-spectrum antibiotics showed an increase in Foxp3+ regulatory T-cells (Tregs) in the cervical and mesenteric lymph nodes, receiving lymph drainage from the eye and the gut, respectively.^[29]^ This finding suggests there was influence on the immune response in the eye and in the gut.

Several studies also provide evidence of the gut microbiota's role in other ocular diseases, thus supporting the presence of gut–eye axis. Zaheer et al^[21]^ studied the CD25KO murine model that exhibit spontaneous features of severe Sjögren syndrome (i.e., dacryoadenitis, sialadenitis, and keratoconjunctivitis). This study revealed that CD25KO mice raised in germ-free (GF) environment have greater corneal barrier disruption, lower conjunctival goblet cell density, and greater lacrimal gland lymphocytic infiltration that progresses to complete gland atrophy compared to conventional CD25KO.^[23]^ Meanwhile, Rowan et al^[30]^ demonstrated the gut–eye axis in mice model with AMD. Mice fed with high-glycemia diet developed AMD features, such as RPE hypopigmentation, RPE atrophy, and photoreceptor degeneration. Higher proportion of *Firmicutes* and *Clostridia* as well as lower proportion of *Bacteroidetes* and *Erysipelotrichi* were also found in mice with greater retinal damage.^[30]^

### The Role of Gut Microbiome in Autoimmune Uveitis

Furthermore, the specific role of gut microbiome in autoimmune uveitis has been linked with the presence of peculiar memory responses toward retinal arrestin and IRBP. Both (retinal arrestin and IRBP) are proteins expressed in a niche location behind a tight blood–retina barrier. The blood retina barrier may only be crossed by activated lymphocytes, this is done by initiating a transient breakdown in the blood–retina barrier via cell rolling, extravasation through venules, and reduced claudin and occludin. Although the mechanism is not quite clear, similar observations have been seen in the CNS.^[20]^ However, in order to activate the specific T-cells, it is necessary to be exposed to the retinal arrestin and/or IRBP. Thus, suggesting presence of mimicry antigens outside the eye leading to retina-specific T-cell activation independent of endogenous retinal antigen.^[19]^ Horai et al proposed that gut microbiota provides signals directly to the retina-specific T-cell receptor thus causing these autoreactive T-cells to trigger uveitis. The study proposed the possibility of (a) gut microbiota mimicking retinal antigens or (b) microbiota as an adjuvant providing innate signals, in which both (a) and (b) mechanisms amplify host immune response in activating autoreactive lymphocytes specific for neuroretina.^[19,31]^ They studied R161H mice, which are designed to develop spontaneous uveitis via the expression of R161 T-cell receptor specific for 161-80 of IRBP.^[19]^ In these mice, activated uveitis-relevant T-cells were apparent in the lamina propria of small and large intestines even before the onset of clinical uveitis, suggesting activation of T-cells in the periphery. This activation step in the periphery is crucial because only then will the retina-specific lymphocytes be able to breach the blood–retinal barrier. Depletion of commensal microbiota in R161H mice, via antibiotic treatment or GF conditions, resulted in significant attenuation of spontaneous uveitis and reduced populations of Th17 cells in the gut lamina propria. Spontaneous uveitis development was associated with increased populations of Th17 cells in the intestinal lamina propria.^[32]^ This supports the findings in depleted R161H and GF R161H where clinical manifestation and Th17 cells of the lamina propria is reduced.^[19,32]^ In an earlier study by Horai et al, it is recognized that T-cell activation in the intestine is independent of endogenous IRBP expression.^[32]^ They crossed R161H mice to Rbp3–/– mice, which lack IRBP expression. The R161H– Rbp3–/– mice did not developed uveitis, due to lack of target antigen in their eyes. However, IRBP-specific T-cells were still found within the mice and were functionally responsive to IRBP. When these activated IRBP-specific T-cells were transferred to native white mice, it successfully induced severe uveitis.^[32]^ These findings suggest endogenous IRBP is not mandatory in activation of IRBP-specific T-cells in R161H mice.^[19,31][32]^

As the role of dysbiosis in ocular autoimmune diseases is further studied, the role of microbe translocation is still less known. Microbial translocation in other diseases have been observed, including rheumatoid arthritis, SLE, etc.^[14,18]^ Microbe translocation to the eye contradicts with the widely accepted concept of a sterile intraocular environment due to the blood–retina barrier. However, a study from Deng et al recently revealed preliminary evidence of disease-specific microbial presence in human aqueous humor, which is previously known to be sterile, in patients with AMD and glaucoma.^[21]^ They also found *Propionibacterium acnes* in most eyes from patients who underwent cataract surgery. This species of bacterium was one of the most common bacteria detected in chronically inflamed eyes after cataract procedure.^[21]^ These early findings favor microbiome's role in the ocular inflammatory states.

### Fecal Microbiota Transplantation (FMT)

FMT is performed by the administration of donor fecal solution into the recipient intestinal tract, which can be done via oral route or direct implantation using colonoscopy. The key mechanism by which FMT may influence disease progression is through repopulating and returning gut microbe colonization into its homeostatic state as well as improving the intestinal tight junction. In murine models following FMT intervention, *Bacteroidetes* (pro-inflammatory) phylum was found to be decreased, while the *Firmicutes* and *Lactobacillus* (probiotic) phylum were increased.^[33,34]^ These conditions enhance gut barrier integrity, limit microbiota and byproduct from entering systemic circulation, hence preventing the activation of inflammatory cascade.^[13,34]^

Desirable outcome of FMT has been reported in numerous studies mentioning various organs, from infection to autoimmune origin, for example, Clostridium dificille infection,^[35,36]^ irritable bowel disease,^[37]^ chronic fatigue syndrome,^[38]^ nonalcoholic fatty liver disease, idiopathic thrombocytopenic purpura,^[39]^ and multiple sclerosis.^[40]^

In ocular diseases, FMT has demonstrated apparent gut–eye relationship in mice model. Ye et al observed a significantly exacerbated experimental autoimmune uveitis (EAU) manifestation after administering FMT from humans with Behcet's disease to B10RIII mice. This was further supported with investigation using RT-PCR, in which they found increased production of inflammatory cytokines including IL-17 and IFN- γ in the spleen.^[28]^ Zaheer et al developed a murine model, CD25 knock out (CD25KO), mice lacking of IL-2 receptor alpha chain (CD25) – which exhibit no IL-2 signaling, lack of Treg cells, and hindered apoptosis of their autoreactive T-cells. This mice model undergoes spontaneous development of severe Sjögren syndrome features such as dacryoadenitis, sialadenitis, and keratoconjunctivitis. They compared clinical manifestations, quantified the expression of T-cell and inflammatory cytokines between the CD25KO raised in GF environment versus the conventional CD25KO. GF CD25KO showed greater corneal barrier disruption, lower conjunctival goblet cell density, and greater lacrimal gland lymphocytic infiltration that progresses to complete gland atrophy, compared to the conventional CD25KO. However, after transplanting fecal slurry contained intestinal microbiota from the conventional C57BL mice to GF CD25KO via oral gavage, the GF CD25KO showed decreased generation of pathogenic CD4 ${}^{+}$ IFN- γ ${}^{+}$ cells, resulting in improved lacrimal gland pathology and greater Goblet cell density, thus shortening the disease duration. The corneal barrier function showed significant improvement, with similar esophagogastroduodenoscopy staining level compared to the Oregon-Green dextran (OGD) dye conventional CD25KO, thus preventing further physiological state disruption.^[23]^

Receiving heavy recognitions from multiorgan system, the study of FMT in relation to ocular diseases in humans is still unavailable, therefore we propose a new insight upon this. A standard criteria for feces donor is not yet defined, however, Amsterdam protocol has been the main reference for this field.^[42]^ Fecal material is gained from healthy donors who meet specific requirements (e.g., the absence of antibiotics consumption in certain duration, no history of intravenous drug use, high-risk behavior, or any infectious, neoplastic, metabolic, autoimmune, or allergic disease).^[42]^ In its frozen state, stool for FMT can be stored for six months without loss of clinical efficacy or bacteria viability.^[43]^ FMT can be delivered through oral consumption, esophagogastroduodenoscopy, nasojejunal tube, nasogastric tube, colonoscopy, or retention enema.^[49]^ Studies upon FMT safety and efficacy are growing and appear to be safe. A review from Smits et al reported from > 3000 FMT at the Centre for Digestive Diseases in Australia and > 200 at the Academic Medical Center in Amsterdam, with no serious adverse events observed for a six-months to two-years follow-up.^[42]^ Currently, there are 114 studies registered at www.clinicaltrials.gov for FMT as therapeutic treatment, and are still recruiting. These findings indicate the feasibility of FMT as a targeted and practical therapeutic agent.

### Probiotics 

Probiotics are live strains of selected microorganisms which when administered in adequate amounts, confer health benefit to the host by improving the gut flora, preventing growth of unwanted pathogens, and improving immunity. Probiotics are resistant to gastric acid, bile, and trypsin, and are still viable to colonize, thus proliferate inside the gut afterward.^[44]^

Probiotics play a role in the immune system through several mechanisms: (1) enhancing the gut chemical and biological barriers via the space-occupying effect, (2) increasing the tight junction protein synthesis between epithelial cells via promotion of mucous glycoprotein secretion, and (3) regulating innate and adaptive immunity via gut-associated lymphoid tissues (GALT). Probiotic and its metabolites possess antigens that are phagocytized by M-cells to form endosomes. The antigen in M-cells are then released and received by dendritic cells, thus presenting them to naive T- and B-cells of the lymph nodes, creating immune responses mediated by TLR, NOD-, NLR, Th1/2, Treg, TGF-beta. As the T- and B-cells turn into different effector subpopulations, they correspond to different immune functions.^[44,45,46]^

Probiotics are commonly used in multiple diseases. However, its usage in ocular autoimmune diseases are still scarce. A study in mice model with EAU by Kim et al showed decreased manifestation of uveitis when given IRT-5 probiotics-mix (a mixture of *Lactobacillus casei, L. acidophilus, L. reuteri, Bifidobacterium bifidum, *and *Streptococcus thermophilus)*. They found decreased numbers of Tregs in the cervical lymph nodes and decrease of CD8+ T-cells in the given IRT-5 probiotics-mix mice.^[45]^ In human studies, topical ocular probiotics have been used in patients with vernal keratoconjunctivitis by Iovien et al.^[47]^ Meanwhile oral probiotics have been used in a study by Miraglia Del Giudice et al, in which *Bifidobacterium* mixture (*B. longum BB536, B. infantis M-63, B. breve M-16V) *improved symptoms in children with seasonal rhinoconjunctivitis and intermittent asthma.^[48]^

Iovien et al demonstrated the use of *Lactobacillus acidophilus* diluted as eye drops to vernal keratoconjunctivitis, resulting in improved clinical outcomes within two to four weeks. *Lactobacillus acidophilus* is suspected to have anti-inflammatory properties via IL-10 and TGF-beta.^[47]^ Probiotics modulate immune responses via stimulation of the Th1 pathway and restoration of T regs leading to improved allergic responses.^[48]^

Zmora et al administered 11 species of common probiotic bacteria (*Lactobacilli, Bifidobacteria, Lactococcus lactic, Streptococcus thermophiles*) to healthy participants for 28 days. They detected the probiotic bacteria in several participants' feces sample, suggesting that transient engraftment was depended on the initial host's microbiome composition.^[49,50]^ Further longitudinal studies are needed to evaluate their efficacy.

##  DISCUSSION

Recent studies supporting the gut–eye axis have revealed more information on the possible pathomechanism of autoimmune uveitis.^[1,3,4,13,15,20,29,30]^ To date, studies targeting the microbiome for therapy of uveitis are still very limited. Among the available treatments for autoimmune uveitis, effective targeted therapy is yet to be uncovered. From this point, the concept of atopobiosis and dysbiosis are elaborated as the etiology or exacerbating factor of autoimmune uveitis.^[19,32]^ Hence, we believe halting these pathomechanisms as a targeted treatment might be a promising solution.

### Atopobiosis/Dysbiosis–Uveitis Relationship

Our hypothesized pathogenesis of gut atopobiosis and dysbiosis in relation to uveitis has been synthesized and demonstrated in Figure 1. Microbes, especially bacteria in the gut lumen communicate with the enterocyte through four possible distinct pathways: (1) recognition of microbe-associated molecular pattern by toll-like receptors (TLR); (2) bypassing the tight junction following an inflammatory state which increases the intestinal permeability; (3) transcytosis via microfold cells; (4) phagocytosis of microbes and antigen presentation by dendritic cells (DC) or APC. In the case of atopobiosis, microbes enter the mesenteric lymph node and gain access to the systemic circulation. The translocated microbe may or may not cause systemic manifestation depending on its dormancy and numbers. The microbe may travel further to ocular endothelial sites and may be able to surpass the blood–retina barrier if it were not intact [Figure 1.5a–1.7a). Although the intraocular environment was long established as a sterile environment, recent preliminary studies reveal microbial intraocular presence. Thus, suggesting the possibility of microbial gut translocation to the eye, warranting the need for ocular microbiome identification to further shed light on a possible causative relationship.^[21]^ In contrast, dysbiosis occurs by DCs, T-cells, and B-cells entering mesenteric lymph node. Some may undergo the cascade of antigen presentation from DC to T-cells and differentiation of B-cells to plasma cells. These cells may also further be present in the lamina propria, in which the retina-specific T-cells may encounter mimicry antigens (such as proposed microbes), leading to activation of retinal-specific T-cells. As these retinal-specific T-cells are capable of passing the blood retina barrier, they may cause local inflammation in the eye [Figure 1.5b–1.7b].

Although much more limited compared to dysbiosis,^[3,13,15,17,25,29,30,51]^ evidence for atopobiosis is found for other organ diseases.^[18,21,22]^ A recent study by Gómez et al^[22]^ has revealed the association of atopobiosis of *P. gingivalis* from dental infection to the placenta, resulting in outcomes adverse pregnancy outcomes (APOs) including low birth weight, preterm birth, preterm premature rupture of membranes, as well as other conditions related to chorioamnionitis. Using 16S rRNA assay, they found the periodontal infection microbes (*P. gingivalis)* in the placenta of women presented with APOs, and not in women with healthy pregnancy. It was hypothesized that the microbe translocates via systemic and placental route, thus activating inflammatory response of the decidual tissue. It then shifted the Th-1 profile balance toward an inflammatory state, mediated by MCP-1 and macrophages. Microbes were found in the placenta; and placental cytokine patterns showing reduced IL-10, IL-17F, and a Th-1 profile which induced macrophage activation by increased MCP-1, were found in these women who clinically presented with APOs.^[22]^ The possibility of a similar occurrence in the eyes was seen by Deng et al.^[21]^ The blood–retina barrier which protects the sterile intraocular environment would typically prevent the passage of hematogenic pathogens. However, staggering recent evidence from Deng et al shows findings of intraocular microbial presence. In their study, a disease-specific microbial signature was found in aqueous humor of patients with AMD and glaucoma.^[21]^ As the aqueous humor has no direct access to the external environment, the possibility of a hematogenic spread via the blood–retina barrier comes into question. Factors affecting the intact barrier such as trauma or local immune responses in particular diseases, such as AMD, may factor in the penetration of the blood–retina barrier. Thus, with current findings of atopobiosis in multiple target organs and presence of intraocular microbes, further investigation is mandated to determine a causative relationship.

As numerous studies^[1][3][4][5][6][11][12][13][15][18][19][20][21][22][23][24][25][26][33,34][35,36][37][38][48][52,53][54][55,56]^ mentioned in this review contribute toward the hypothesis of gut dysbiosis in relation to ocular autoimmune uveitis, it is only wise that we explore further the possibility of targeting gut microbiome as a mean to alter clinical manifestation. As current therapy for autoimmune uveitis mainly rely on to suppress symptoms, it is appealing to find alternative targeted therapy with less adverse effects.

### Challenges for Future Studies

Up to this date, there is still no established cut-off of the “normal gut microbiome”, as it is highly affected by numerous internal and external factors, for example, genetics, age, ethnicity, diet, geographical region of domicile; making it individually distinct. A metagenomic characterization in 2018 revealed that every anatomical region of mammalian gastrointestinal tract demonstrates distinct oxygenation level, pH, host-derived antimicrobial and transit time.^[12,28]^ In 2019, the recent shotgun metagenomic characterization of gut microbiome successfully demonstrated bacteria to the species- and strain-level classification, also fungal residents' characterization.^[12]^ Those aspects together influence the local microbiome assemblage, adding another question in the characterization of the “normal gut microbiome”: which section should we refer to when defining normal gut flora?^[28]^

The two main methods used for microbiome characterization are the 16S rRNA assays and the shotgun metagenomic, which enables microbiome identification to the genus-level and species- and strain-level, respectively. However, the shotgun metagenomic requires extravagant cost and advanced bioinformatics.^[12,30]^ Both approaches mainly identify bacteria and recently fungi. These suggest potential diagnostic tools for further studies. Further techniques capable of evaluating the functional status of microbes and other non-prokaryotic constituents of the gut are also needed.

Further studies on the mechanism of atopobbiosis in the gut–eye axis should involve a series intraocular microbiome profiling. The intraocular microbiome profiling should include ocular diseases such uveitis and AMD, followed by profiling of the corresponding gut microbiome within the same test subjects. Moreover, microbiome profiling of the aqueous humor and gut in test subjects receiving active interventions to the gut microbiome such as FMT and probiotics is suggested for future studies. Thus, elucidating the complex relationship of the gut–eye axis.

Current studies targeting the microbiome for therapy of uveitis are still very limited. Studies utilizing FMTs in uveitis patients have only progressed to the use of mice treated with FMTs from human samples, which has been done by Ye et al with 11 mice subjects.^[28]^ Present studies characterizing the gut microbiome of uveitis patients are not only small in sample size, but also mainly focuses on patients with Behcet disease and Vogt-Koyanagi-Harada syndrome, which are only a fraction of uveitis patients. Once we can agree on a consensus defining the “normal microbiome”, how to detect it, and characterize the gut microbiome of heterogenic uveitis subjects, only then we can gain robust evidence to proceed to clinical trials for FMT and probiotics in humans.

Future longitudinal studies with microbiome sequencing involving greater number of autoimmune patients are expected to elucidate how atopobiosis and dysbiosis influence the microbiome profile. Clinical trials for FMT and probiotics are expected, particularly seeing this might come as a promising solution to mitigate autoimmune uveitis, to shorten disease duration, also to prevent further physiological state disruption.

##  Financial Support and Sponsorship

Nil.

##  Conflicts of Interest

There are no conflicts of interest.
